# Bombesins: A New Frontier in Hybrid Compound Development

**DOI:** 10.3390/pharmaceutics15112597

**Published:** 2023-11-07

**Authors:** Pawel Serafin, Patrycja Kleczkowska

**Affiliations:** 1 Military Institute of Hygiene and Epidemiology, 01-163 Warsaw, Poland; pawelserafin1@wp.pl; 2 Maria Sklodowska-Curie, Medical Academy in Warsaw, Solidarnosci 12 Str., 03-411 Warsaw, Poland

**Keywords:** bombesin, bombesin receptors, hybrid development, efficacy, biological properties

## Abstract

Recently, bombesin (BN) and its analogs have attracted much attention as excellent anticancer agents because they interact with specific receptors widely distributed on the surface of various cancer cells. However, their biological properties proceed far beyond this, given a broad spectrum of activity. Bombesin receptor ligands are effective drugs for the treatment of rheumatoid arthritis or gastrointestinal diseases. However, most diseases are complex, and the use of polytherapy may lead to pharmacokinetic and pharmacodynamic drug–drug interactions, resulting in side effects. Therefore, there is a need to develop effective compounds that also contain BN or its analogs, which are combined with other structural entities, thus generating a so-called hybrid drug. Hybrid drugs that contain bombesin pharmacophore(s) may be proposed as a solution to the problem of polytherapy or the lack of an effective cure. Such structures have now demonstrated the desired efficacy, though information on these aforementioned compounds is relatively scarce. Therefore, our paper aims to encourage researchers to focus on bombesins. Herein, we indicate that the hybrid approach should also be firmly applied to bombesins and the BN receptor family. This paper’s structure is divided into two main sections demonstrating bombesins and their properties, as well as recent data on bombesin-based hybrid compounds and their potential usefulness in medicine. Overall, it refers to the discovery and synthesis of modified bombesin-based hybrid compounds.

## 1. Introduction

**Figure 1:**
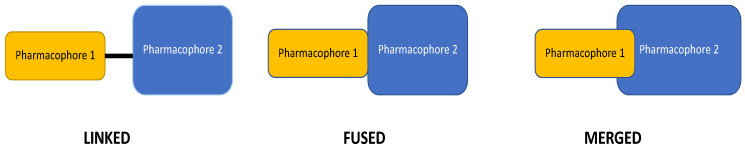
Different types of molecular hybridization.

Hybrid compounds are potential drug candidates that are useful in the treatment of various complex diseases or when polypharmacotherapy is required. They consist of functional parts covalently assembled into a single molecule using strategies that include splicing or pharmacophore fusion and the use of cleavable/non-cleavable linkages (Figure 1) [1,2]. Hybrid compounds (also known as chimeras and designed multiple ligands (DMLs)) aim to improve a drug’s pharmacokinetic and pharmacodynamic profile while enhancing its biological activity and reducing clinically relevant side effects compared to their parent molecules. These features are due to chimeras’ ability to interact with a single target or multiple related and unrelated molecular targets.

There are many examples of hybrid structures involving pharmacophores that differ in their mechanism of action. They may consist of different classes of substances, including organic molecules, polypeptides, etc. Some of them are a combination of opioids with other bioactive molecules involved in pain signaling (e.g., melanocortin, substance P, neurotensin), making them suitable for the treatment of pain. Other entities combine compounds with potent effects in the treatment of cancer and Alzheimer’s disease and even to combat parasites (e.g., curcumin-quinolone hybrids, coumarin-indole hybrids, imipramine-quinacrine) [3,4,5,6].

**Figure 2:**
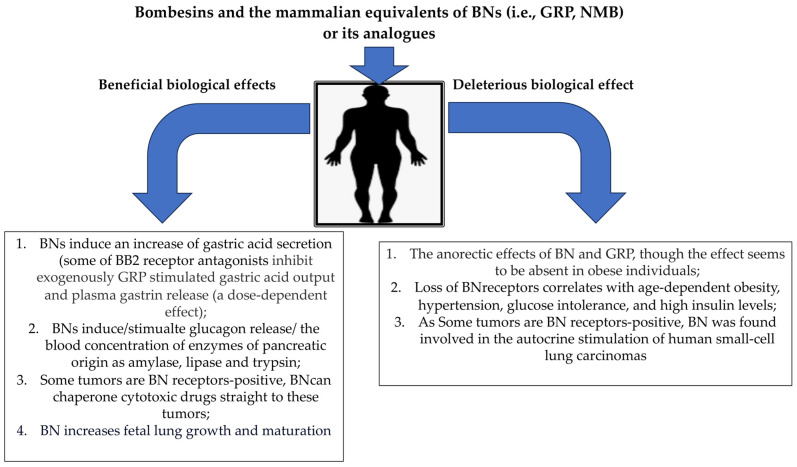
Examples of some of the clinically confirmed beneficial and deleterious biological effects of bombesins in humans.

Recently, bombesin (BN) and BN-like peptides have attracted considerable research interests because they have revealed a broad pharmacological spectrum (Figure 2). Therefore, combining such ligands into a hybrid structure could serve as valuable drug candidates.

## 2. Bombesins and Bombesin Receptors

### 2.1. Mammalian Bombesins

Bombesin (BN) is a tetradecapeptide originally isolated from the skin of an amphibian, *Bombina bombina* [7]. Importantly, and subsequently, two bombesin-like peptides, the gastrin-releasing peptide (GRP) and neuromedin B (NMB), were identified in extracts of mammalian gastric tissues and the spinal cord, respectively. The amino acid sequence analysis demonstrated that all three bombesins possess almost identical carboxyl terminus with a slight difference between phenylalanine (Phe) in NBM and leucine (Leu) in GRP and BN (Table 1). Also, when analyzing the carboxy-terminal nonapeptide fragment of BN and GRP, it can be noticed that these two peptides are identical except for the single substitution in GRP of histidine for a glutamine residue at the eighth position starting from the C-terminus.

Erspamer and colleagues, as well as other researchers, reported bombesin and the two newly discovered peptides as compounds with the ability to exert multiple biological effects when administered in mammals [7,8,9], including its inhibitory action on gastric and acid secretion, body temperature control, the mediation of pruritus, smooth-muscle contractions, or even the stimulation of either normal or neoplastic tissue growth (Table 1) [10,11,12,13].

Indeed, for instance, BN is well-known for its great potency in suppressing food intake [14]. In addition, a BN-induced reduction in ethanol consumption was observed. In line with this, Deschodt-Lanckman et al. [15] reported that BN stimulated amylase secretion from the mammalian pancreas in vitro. Also, the secretion of cholecystokinin CCK from the I cell of the small intestine, as well as other compounds, including the vasoactive intestinal peptide or even insulin, were observed for the peptide [16,17]. BN was also demonstrated to possess mitogenic activity, especially in the case of Swiss 3T3 cells, and can act as an autocrine growth factor for small-cell lung cancer (SCLC) [18,19,20]. Furthermore, it was reported that BN may act as a potent antioxidant, as Assimakopoulos and colleagues [21,22] showed BN to reduce intestinal oxidative stress (via decreasing intestinal lipid peroxidation) in an animal model of experimental obstructive jaundice and after partial hepatectomy. Several papers present BN to produce a hypertensive response after its central and systemic administration [23,24]. Others provide evidence that BN is characterized by its ability to significantly increase locomotor, rearing, and grooming activities, which are time- and dose-dependent. Importantly, these behavioral effects were blocked in studies by neuroleptics such as haloperidol, which further suggests the possible interaction of BN with a dopaminergic system [25]. In 2008, BN was also found to stimulate the expression of factors involved in the wound-repair process [26].

Similar to BN, GRP was also found to mediate some effects. Recent studies suggest an important role for GRP and, thus, the bombesin type 2 receptor (BB2 receptor) in several new areas, including the mediation of pruritus [27]. This was further confirmed by the use of BB2 receptor antagonists, which resulted in a significant decrease in GRP-induced scratching behavior. Noteworthy, GRP-induced scratching behavior was not associated with a pain sensation [28]. This neuropeptide was also indicated to be directly associated with rheumatoid arthritis pathology by influencing the production of proinflammatory cytokines [29]. Furthermore, GRP plays an important role in the circadian rhythm through the suprachiasmatic nucleus [30].

In addition to the above-mentioned discoveries, GRP was also revealed as a beneficial compound. Firstly, it has been reported that GRP can enhance memory retention in vivo [31]. However, some contradictory reports can also be found. Flood and Morley [32] provided information that this effect may vary depending on the animal model or route of drug administration; for instance, GRP enhanced retention in sham and non-operated mice but not in vasectomized mice. Moreover, together with BN, GRP reversed scopolamine-induced amnesia [32,33]. Other beneficial actions of GRP, as well as its agonist analogs, include but are not limited to the following: (i) the prevention of enteric ganglia atrophy in the small bowel [21], (ii) a reduction in fear in the fear-related paradigm in vivo [34,35], (iii) the restoration of penile reflexes and ejaculation after castration in vivo [36], etc.

These beneficial effects have also been demonstrated in studies indicating the possible antimicrobial activity of the BN family. In fact, it has been suggested that frogs secrete bombesins either as antimicrobial peptides or toxins, which may protect them from bacterial infection and/or predators in terrestrial adaptation. Obviously, such activity is of great value for humans, especially in view of the increasing bacterial resistance to numerous antimicrobial drugs. In this context, BN itself was found to play a role in maintaining respiratory defenses against both viruses and bacteria by inducing effects on mucosal immunity [37]. Other studies provided results for GRP analogs. A good example is the compound RC-3096, which is a selective BB2 receptor antagonist that has been shown to protect the lungs against a locally administered *Escherichia coli* endotoxin [38].

NMB, a decapeptide isolated from the porcine spinal cord, together with its corresponding bombesin type 1 receptor (BB1 receptor), has an essential role in thermoregulation in parallel with GRP/BB2 [9,39]. Also, as shown earlier, NMB—similar to GRP—affects stress response [40]. Indeed, many effects mediated by NMB overlap with GRP, though some of them are specifically related to the action of NMB; these include the contraction of the smooth muscle and growth effects in various normal and neoplastic tissues [41,42,43]. NMB was also implicated in pain transmission [44,45]. Mishra and colleagues [45] reported that the peripheral (i.e., intraplanar, i.pl.) injection of the neuropeptide resulted in a neurogenic inflammatory response, exposed as local swelling and thermal and mechanical hypersensitivity. Likewise, pretreatment with an NMB receptor antagonist decreased mustard oil-induced swelling and hyperalgesia. However, later findings of Wan et al. [46] indicated that this effect was not mediated by BB1 receptors.

Several unrelated studies suggest a new role for NMB as a stimulatory peptide of the hypothalamic–pituitary–gonadal (HPG) axis, which is likely mediated via the hypothalamic gonadotropin-releasing hormone [47,48,49]. Recently, NMB was also reported to be involved in the pathogenesis of vascular smooth muscle cell calcification through the modulation of high phosphate-induced calcification [50]. Finally, this neuropeptide has been proven to produce mitogenesis in adipocyte 3T3 cells, which leads to a decrease in obesity [51].

All these biological actions resulted from the existence and, thus, the peptide’s interaction with specific receptors. Three members of the mammalian bombesin receptor family have been cloned so far, each from the GPCR family; the BB1 receptor is also a mammalian NMB-preferring receptor (neuromedin B receptor), BB2 receptor (a GRP-preferring; GRP receptor), and an orphan receptor designated as the bombesin receptor subtype-3 (BB3) [52]. It is noteworthy that amphibian bombesin subtype 4 (bombesin-4) receptors and a unique Bn-R from chickens termed chBRS-3.5 were also reported [53]. These receptors were demonstrated to possess either a high affinity for BN rather than GRP or a moderate affinity for BN but low for GRP and NMB, respectively [39]. Nevertheless, in the case of the chick brain receptor, no mammalian equivalent to this receptor has been described so far.

Several studies regarding bombesin and mammalian bombesin-like peptides have revealed that these compounds are widely distributed in the central nervous system (CNS) and peripherally. Each peptide is present mainly in the brain and gastric tissues. However, they can exert diverse effects based on localization. For example, bombesin-like peptides were found to dose-dependently stimulate gastrin and gastric acid secretion when administered peripherally, while, when given directly to the brain, BN, and GRP behaved as potent inhibitors of gastric acid secretion [54]. This is true both for humans and animals (in particular rats, cats, and dogs) [55,56,57]. Intriguingly, these effects were mediated through a different mechanism, as the inhibitory action resulting from intracisternal BN was found to be associated with the production and release of nitric oxide (NO) [58]. By contrast, in the stimulatory effect as a consequence of, e.g., intravenous BN administration, gastrin-dependent mechanisms were suggested [59,60].

Apart from these peptides, their receptors are also distributed throughout the body. BB2 receptors are highly expressed throughout the brain, including the hypothalamus and amygdala, while BB1 may be found in a more restricted fashion, particularly in the olfactory and thalamic areas [61]. In the periphery, BB2 is also highly concentrated in the stomach, and pancreas and is slightly expressed in the colon, breast, lungs, or prostate [62]. Regarding BB1 receptors, the highest levels of BB1 mRNA were detected in the testis and stomach whereas in rodent peripheral tissue, significant expression was found in the esophagus, intestine, testis, and uterus. In contrast, the BB3 receptors are limited to the hypothalamus, cerebral cortex, and thalamus in the CNS and gastrointestinal tract in the periphery [63,64]. Unfortunately, due to a lack of information available on the native ligand of the BB3 receptor, its distribution and the possible effects mediated by its activation are less well-studied.

### 2.2. Amphibian Bombesin-like Peptides and Their Receptors

Bombesin-related peptides can be distinguished based on their origin in mammalian and amphibian peptide structures, although they share homologous structures. As presented previously, the first group includes both the above-mentioned gastrin-releasing peptide (GRP) and neuromedin B (NMB). Whilst, in amphibians, three families of bombesin-like peptides have been characterized: the bombesins (bombesin and alytesin), the ranatensins (ranatensin, ranatensin C and R, and litorin), and the phyllolitorins (Table 2). The phyllolitorin group consists of phyllolitorin, [Leu^8^]phyllolitorin and [Thr^5^,Leu^8^]phyllolitorin. It is noteworthy that the bombesins family covers various structurally related bombesins based on the type of frog, the skin of which secrets the peptide, e.g., bombesin-RS from *Rana shuchinae* [65], BR-bombesin from *Boana raniceps* [66], and bombesin-SV from Sanguirana variants [67].

As presented in Table 2, all these have pyroglutamyl N-terminal and C-terminal octapeptide residues (Phe for Leu at the penultimate position of ranatensin, phyllolitorin, and litorin). BN and alytesin differed in only 2 of their 14 amino acid residues (Gln → Gly and Asn → Thr, respectively), and both peptides had a marked carboxy-terminal sequence homology with ranatensin.

Unfortunately, concerning amphibian bombesin-like peptides, the number of papers characterizing their biological properties is rather scarce, and most of the reports are related to studies performed in the last century, between 1970 and 1990.

Litorin was first isolated from the extracts of the skin of the Australian frog Litoria aurea. As demonstrated in Table 2, litorin exhibits stronger affinities toward both BB1 and BB2 receptors than BN. This activity is reflected in the litorin-induced effect on the smooth muscle, as this peptide was found to be more potent than BN. Although litorin resembles the pharmacological effects made by BN, Endean et al. reported that the actions of litorin are more rapid in onset and disappearance than those observed for BN, either in vitro or in vivo [68]. In line with this, litorin stimulates gastrin and gastric acid secretion in dogs as well as prolactin secretion in rats [69,70]. Moreover, it reduces plasma TSH levels in a dose-dependent manner, possibly through its interaction with a serotonergic system [71]. When administered intracerebroventricularly, i.c.v. (by pulse or continuous flow) into rats in the angiotensin II-induced drinking model, litorin behaved differently. In fact, after pulse i.c.v. injection, this bombesin-like peptide did not significantly affect drinking, and this effect was independent of the dose administered. In contrast, when given via i.c.v. infusion, litorin showed a marked inhibitory effect. However, in the case of water deprivation-induced drinking, it was found to be almost as active as BN in its inhibitory action of water intake [72].

Bombesin-like peptides were widely demonstrated to selectively inhibit caloric intake. This was also true for litorin, which produced similar suppressions of food intake [73]. Furthermore, when administered i.pl., it suppressed the intake of 5% ethanol [74].

Another amphibian bombesin-like peptide, ranatensin, due to some structural similarity to hypotensive peptides such as eledoisin, was widely studied in the aspect of its potential impact on blood pressure and heart rate. In this context, Geller et al. [75] presented ranatensin activity in various animals (i.e., dogs, rabbits, rats, guinea pigs, or even monkeys) and indicated that this novel peptide influences blood pressure differently. Indeed, while ranatensin lowered blood pressure in monkeys after its intravenous administration, the blood pressure response to the peptide observed in rats was variable and, in part, could be related to the basal level of pressure [75]. In contrast, blood pressure in the dog and rabbit was significantly increased; this effect was not altered by atropine or propranolol, suggesting a direct peripheral vasoconstrictor action.

Like litorin and BN, ranatensin revealed its potency and efficacy in terms of its ability to cause the residual stimulation of amylase release [76]. However, the stimulation of amylase release in mouse pancreatic acini appeared to differ from its ability to cause the direct stimulation of amylase release in guinea pig pancreatic acini. In fact, in mouse pancreatic acini BN, litorin and ranatensin were equipotent in their abilities to cause the direct stimulation of amylase release, whereas in guinea pig pancreatic acini, ranatensin was three times less potent than BN and litorin was 10 times less potent than BN [77].

The spectrum of biological activity of alytesin is not wide, and some pharmacological studies are still in progress. Nonetheless, it was found to cause a hypertensive action in dogs with marked tachyphylaxis. Also, an intense stimulatory effect on rats as well as the guinea pig colon was noted [7]. In 2008, Cline and colleagues [78] showed alytesin to possess anorexigenic activity after its central (i.c.v.) or peripheral (i.p.) administration to chicks.

The pharmacological profile of phyllolitorins is similarly poor, as most studies focus on other BN compounds that have potent biological activities. Phyllolitorin differs from the parent peptide litorin by an amino acid substitution in position 7 (a serine residue replacing histidine). However, when looking at phyllolitorin analogs, other amino acid substitutions can also be observed (Table 2). Nevertheless, this group of BN-like peptides is mainly known for its ability to induce excessive grooming, although, in some cases, shorter in duration than that induced by BN. Moreover, as demonstrated by Negri and colleagues, phyllolitorin-induced antidipsogenic activity is structure-dependent. In fact, while [Phe^8^]phyllolitorin was completely inactive, the threonine-substituted phyllolitorin ([Thr^5^,Leu^8^]phyllolitorin) displayed the same effect as BN [79]. Overall, several studies revealed that phyllolitorins may elicit the same panel of effects as BN, including cell proliferation as well as hypothermia [80,81].

### 2.3. Bombesins and Receptor Targets Other than Bombesin Receptors

Due to the structural similarities of bombesin peptides, they were found to interact with specific bombesin receptors (Table 1 and Table 2) despite possessing different affinities. However, these targeted receptors are not the only receptors the peptide can bind to. Some papers demonstrate that BN- and bombesin-like peptide-induced behavioral effects are mediated by the relationship with a dopaminergic receptor system. One of the first to implicate the existence of a relationship between dopamine (DA) receptors and BN was Merali. Together with his group, they first reported that centrally mediated BN effects can be blocked by the use of haloperidol and fluphenazine, both potent dopamine DRD2 receptor antagonists [25,82]. These findings were further confirmed by Van Wimersma Greidanus, who stated that BN and other neuropeptide-induced grooming is inhibited by the dopamine receptor blockade [83,84]. Intriguingly, similar findings were provided for ranatensin. Indeed, in 1991, Zhu et al. [85] suggested that the in vivo ranatensin-induced pain-relieving effect may result from dopamine neurotransmission, as it was attenuated by pretreatment with a DRD2 antagonist—sulpiride. Recently, our group has also shown detailed information on the potential and close connection between the ranatensin and DA system [86]. Using a radioligand binding assay, we confirmed that ranatensin may effectively bind and activate DRD2 receptors. This is consistent with other reports suggesting that BN-like peptides act at reward sites in the brain through the modulation of dopamine and/or GABA activity. Moreover, it was also shown that the direct injection of bombesins in the nucleus accumbens (NAcc) stimulated DA release and that this effect was inhibited by DA receptor antagonists [87]. Another study showed the involvement of BB2 in mediating memory regulation. This study showed that BN-induced memory enhancement was observed as a result of co-infusions of stimulators of the dopamine DRD1/DRD5 receptor (DRD1)/cAMP/PKA pathway, namely the DRD1 agonist SKF 38393, the adenylyl cyclase activator forskolin, and the cAMP analog 8-Br-cAMP [88].

Apart from peptide involvement, BN receptors were also found to modulate the activity of neuropeptide systems other than BN. This is true for BB1 receptor subtypes, which were presented to influence the activity of the serotonergic (5-HT) system. The 5-HT system is well-known to be greatly involved in various behavioral processes, especially anxiety, and responses to stress [89,90]. Therefore, given the aforementioned information on BN and bombesin-like peptides’ contribution to such behavior, a possible reciprocation between these two systems is obvious. Merali et al. and other research groups [91,92] proved that BB1 receptors were functionally expressed in 5-HT neurons in the dorsal raphe nucleus, and their ligand-mediated activation, leading to an increase in 5-HT neuronal firing, which resulted in the upregulation of 5-HT. Other reports revealed that the activation of somatostatin receptors, mainly somatostatin receptor type 2 (SSTR2), as a consequence of somatostatin release is observed in the BN-induced inhibition of gastric acid secretion in vivo. In line with this, BN antisecretory effects were also abolished by the pharmacological blockade of SSTR2 with the somatostatin analog PRL-2903 in wild-type animals [93].

There are several scientific papers demonstrating a so-called “crosstalk” between BN and its receptor system and other receptors. A good example is a paper provided by Liu et al. [94], who demonstrated that the BB2 receptor may interact with a μ-opioid receptor (MOR) isoform 1D, thus functioning as an itch receptor that mediates opioid-induced pruritus in the human spinal cord. Also, Rivier and co-workers [69] reported that BN-induced growth hormone release was reversed by naloxone, thus suggesting an opioid-dependent mechanism of action. Gmerek and Cowan [95] reported that OP receptor agonists act through κ-opioid receptors (KOR), affecting BN-induced scratching in rats. Others provided information on a possible interaction with the orexigenic neuropeptide Y system (particularly in chicks) [96].

Since bombesin-like peptides have some structural similarities with neurokinins, they also appeared to exert similar effects.

Neurokinins, for which substance P (SP; H-Arg-Pro-Lys-Pro-Gln-Gln-Phe-Phe-Gly-Leu-Met-NH_2_) [97] is known as the main tachykinin neuropeptide family member, together with neurokinin receptors, contribute to the pathogenesis of several diseases and states, including cancer, itching or neurogenic inflammation. They are also involved in the stimulation of the intestine as well as the production of IL-6, IL-8, and prostaglandin in rheumatoid arthritis patients [98].

Regarding the abovementioned, in 1988, Regoli et al. [99] discovered BN to be responsible for the inhibition of vascular smooth muscle tone, though this effect was weaker than that observed for SP. At that time, these data suggested that BN may act through the activation of neurokinin 1 receptors (NK-1) [99]. Nevertheless, later findings produced the opposite result, as Sakurada and co-workers demonstrated spantide (an NK-1 receptor antagonist) without any significant effect against the licking, biting, and scratching response induced by BN [100]. Research conducted by Moura et al. [101] showed that GRP can affect the secretion of thyrotropin (TSH). They noted that the administration of SP increased TSH secretion in Wistar rats. However, this effect was not observed when SP was co-administrated with GRP, thus proving that GRP blocked the stimulatory effect of SP on TSH secretion.

**Figure 3:**
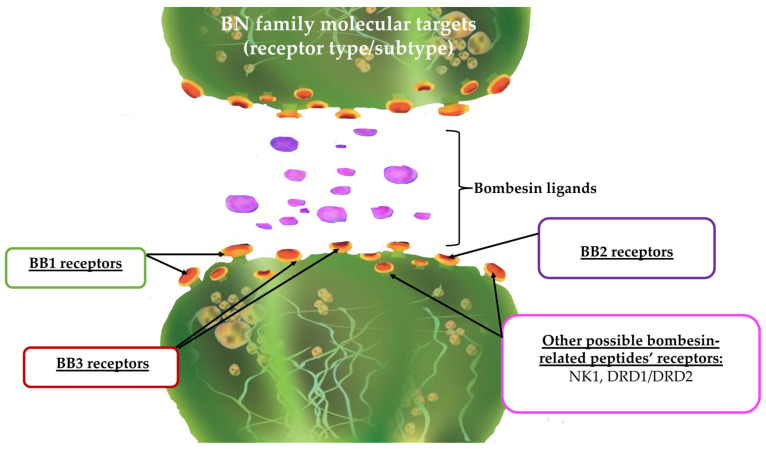
Molecular targets (receptors) for the BN family; BB1—bombesin 1 receptor subtype (neuromedin B receptor), BB2—bombesin 2 receptor subtype (gastrin-releasing peptide (GRP) receptor), BB3—bombesin 3 receptor subtype, NK1—neurokinin 1 receptor, DRD1/DRD2—dopamine 1 receptor subtype and dopamine 2 receptor subtype, respectively.

Such a discovery of existing additional targets (Figure 3), for which the relationship between BN and its related peptides may occur, is of great importance. The effect observed for both the BN, as well as bombesin-like peptides, may result from additional, either direct or indirect, relations of the BN system with other neurotransmitters and their receptors. For instance, dopaminergic receptors as well as NK receptors are strongly involved in many pathological states, including cancer [102]. Therefore, in this aspect, the role of the BN family should also be expanded to tumors expressing such exemplary receptors.

## 3. Hybrid Approach and Bombesins

**Figure 4:**
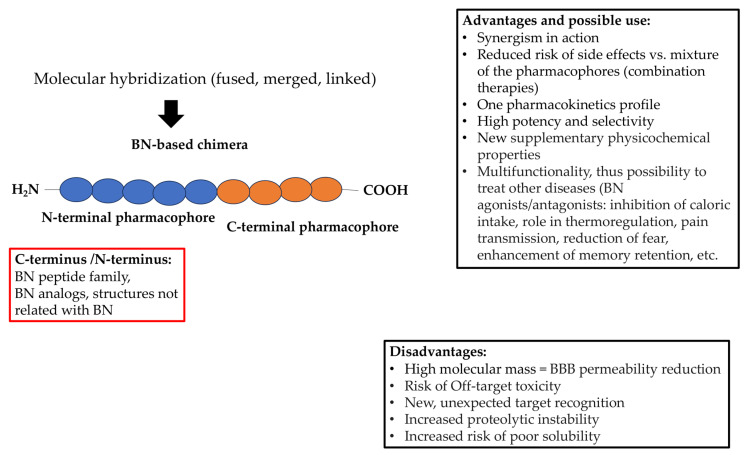
Possible advantages and disadvantages of BN-based hybrid drugs. The red box indicates potential hybrid compositions, as C- or/and N-terminus can be replaced by BN or its analogs as well as other structures with a completely new molecular target.

Unfortunately, the hybrid approach to the synthesis of bombesin-based compounds remains barely perceptible. This negligible lack of interest should be changed immediately, as the BN family possesses numerous desirable properties already mentioned. Indeed, the conjunction of BN or one of its family members or even an analog with another biologically active component can obviously bring many benefits (Figure 4). Moreover, it has been found that BN and BN-like peptides can occupy either the N- or the C-terminal pharmacophore of the chimeric structure to achieve a potentially therapeutic effect without losing activity. However, the prudent design of such molecules should be applied, especially when taking into consideration the fact that BN and its analogs may produce different effects, including adverse ones, depending on their agonistic or antagonistic action on the receptor. Since the BN family also includes peptides, it is also important to preserve key amino acid fragments that are actually responsible for biological activity. It is noteworthy that similar requirements have been identified for opioids and their interaction with opioid receptors. Indeed, it has been proposed that the N-terminal tetrapeptide sequence of endogenous opioid peptides carries the “message” domain, which is responsible for triggering the opioid effect. By contrast, the C-terminus plays an “address” role in determining selectivity for different opioid receptor types [103].

Of course, many attempts to develop effective and safe BN-containing hybrid drugs are to be expected, and this may be largely unsuccessful. This can result from our poor understanding of the behavior of a new molecule, which may act differently than expected based on the activity of the individual fragments of which it is composed. Also, considering that for most hybrid compounds, the molecular weight is about the value given in Lipinski’s rule of five (i.e., >500 Da), a different formulation of the compound might be required in order to achieve its molecular target, and thus, be potentially useful, especially in terms of oral bioavailability [104]. This formulation may additionally be required if the low enzymatic stability of the compound is observed.

Nevertheless, as stated below, several groups have undertaken work to create BN-composed compounds as new drug candidates. Interestingly, the results obtained clearly indicate that the hybrid approach can be successfully used for the BN family. Hence, they may encourage many researchers to turn to bombesins as new chimeric building blocks that deserve attention.

### Bombesin-Based Hybrid Compounds and Their In Vitro and/or In Vivo Efficacy

Although the hybrid strategy in drug development is not new, the literature does not provide much information on hybrid compounds with a bombesin-related pharmacophore. Nevertheless, one of the first conjugates found in the literature is the bombesin saporin. In this case, saporin, a ribosome-inactivating protein known for its ability to induce cell death, was combined to eliminate a specific neuronal subpopulation expressing BB2 in the central nervous system. Several important effects were observed as a result of this combination. Adult male C57BL/6 mice given an intrathecal administration of this hybrid compound showed promise in reducing the number of scratching episodes and attenuating the heat response but did not affect deleterious mechanical responses when administered intrathecally [45,105,106].

Bombesin may also have other potential applications, especially in microbiology. Since the existence of the antibiotic resistance crisis, which endangers the efficacy of clinically available antibiotics, and the lack of development of new drug candidates to address this challenge, BN hybrids may be the proverbial light at the end of the tunnel. In this aspect, Liu [107] was the first to combine bombesin with an antimicrobial peptide (AMP)—magainin II—known also for its antitumor effects against solid tumor cells [108,109]. This approach resulted in a magainin II-bombesin conjugate (named MG2B) [108]. Unfortunately, although this structure contains an AMP, its efficacy was tested in vitro using cancer cells, not pathogens. Nevertheless, MG2B was found to selectively induce cell death in MCF-7 breast cancer cells in vitro and A375, a human melanoma cell line, with the IC_50_ ranging from 10 to 15 μmol/L, which was much lower than the IC50 value of magainin II when administered solely. Moreover, this chimera demonstrated site-selective cytotoxicity, as it is bound to tumor cells with greater affinity than to normal cells. It is noteworthy that, based on this study, it was indicated that a BN pharmacophore with the receptor-binding domain is crucial for the cytotoxic effect exerted by the compound; a BN analog lacking the specific amino acid fragment failed to increase the cytotoxicity observed.

Recently, our group also presented a new BN-based chimera that combines both opioid and ranatensin-like pharmacophores, namely LENART01. This compound is highly active against various strains of *Escherichia coli* in vitro, particularly K12 and R2-R4, at concentrations much lower than those of antibiotics (e.g., ciprofloxacin, bleomycin) [110]. For instance, the 200 μM of LENART01 proved to be almost equally active against the K12 strain as microbial inhibitors were administered at higher concentrations (10 mM/mL). Interestingly, the observed toxic effect of LENART01 exerted on model strains of *E. coli* was found to be strictly dependent on an opioid pharmacophore. Indeed, the inhibition of LENART01 (100 μM/mL) with NLX resulted in a significant reduction in MIC values by almost half for all model strains K12, R2-R4 (**** *p* < 0.0001).

Considering these above-mentioned values, such new bombesin-based structures may serve as a prototype of a new type of antimicrobial molecule that is much more potent than AMPs, which remains the most promising candidate for overcoming rapidly growing antimicrobial resistance. It is noteworthy that while AMPs require specific parameters that are determinants of the spectrum of the peptide activity and its efficacy (e.g., the ratio between hydrophobic and charged amino acids, chain length, etc.) [111], the construction of a bombesin-based hybrid is simple and such physicochemical elements are not required and do not have to be fulfilled. Importantly, by combining BN with another pharmacophore, it is possible that it can act at different targets to reduce the unpleasant effects resulting from the disease but also its origin. A good example is inflammation caused by bacteria, which can be healed by both bombesin-targeting bacteria and opioids acting at peripheral opioid receptors, thus leading to reduced pain and inflammation [112].

**Figure 5:**
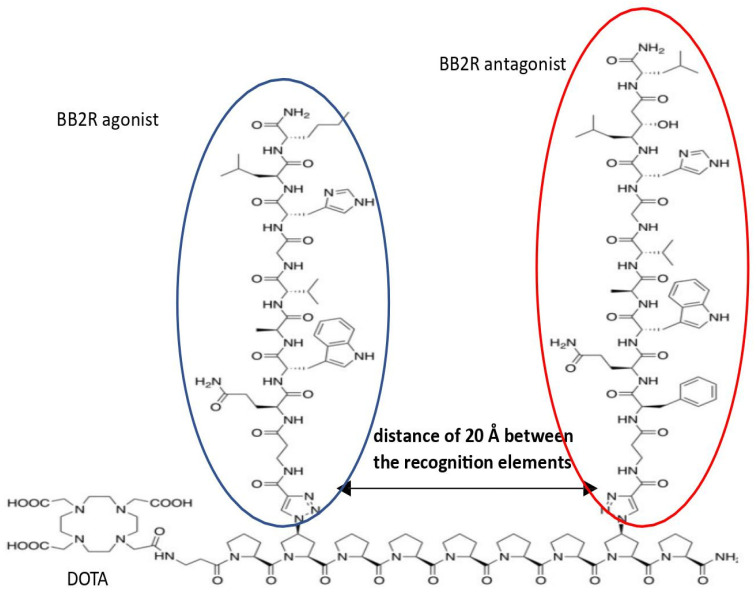
Representative hybrid compound combining ago- and antagonist ligands at BN receptors designed by Kroll et al.

Bombesins are involved in the development and occurrence of various pathological conditions. However, it is fair to say that, ultimately, only cancers have gained much attention from the perspective of BN-based hybrids. Since BNs have revealed their properties as potent compounds, which are useful for molecular imaging and targeted therapy, much work has gone into the synthesis of radio-labeled ligands bound to bombesin receptors. This includes chimeric bombesin structures. In 2013, Kroll and colleagues [113] demonstrated in vitro and in vivo results for novel hybrids containing bombesin-derived BB2-related antagonists and/or agonists bound to molecular scaffolds of oligoprolines at specific distances from each other (Figure 5). In the PC-3 prostate cancer cell line, all compounds showed a high cellular uptake compared with non-hybrid ligands. However, only one, named hybrid 2, had the best characteristics in terms of uptake by the tumor, as well as the wash-out time; this effect was observed in PC-cells xenografted in nude mice [113].

Santos-Cevas [114] designed a hybrid combination consisting of a peptide derived from HIV Tat and bombesin ((99m)Tc-N(2)-Tat(49-57)-Lys^3^-bombesin; (99m)Tc-Tat-BN), which was evaluated in prostate cancer cell lines, but also in breast cancer cell lines MDA-MB231 and MCF7 in vitro. An interesting combination was also carried out by Begum et al. [115], who combined an R9-K(GALA)-BN(6-14) peptide targeting BB2 with a phospholipid oligonucleotide delivery system (1:1 1,2-dioleoyl-sn-glycero-3-phosphoethanolamine DOPE and 1,2-dioleoyl-3-trimethylammonium-propane DOTAP). This new compound was characterized by a significant increase in the expression of BB2-targeted genes in cancer cell lines, especially in the prostate cancer cell line PC-3 [115]. Therefore, a suitable candidate was introduced to transport both pDNA and siRNA into cell lines with BB2 overexpression.

Another example of a hybrid structure in which at least one pharmacophore is BN, or its analog is a cytotoxic compound called AN-215. In this case, 2-pyrrolino-doxorubicin-14-O-hemiglutarate was combined with the BN(7-14) fragment at its N-terminus, and its anticancer activity was evaluated on human pancreatic cancer FPAC-1 and human small-cell lung cancer (SCLC) DMS-53. It is noteworthy that the intravenous administration of this chimera at a dose of 200 nmol kg^−1^ has lower in vivo toxicity compared to the corresponding cytotoxic radicals [116]. This was also confirmed by Kiaris et al. [117].

An interesting combination was made in 2019 by Gibbens-Bandala and colleagues [118], who designed, synthesized, and evaluated the in vitro and in vivo biological activity of a first-line drug for the treatment of solid tumors—paclitaxel cross-linked with BN using poly(lactic acid-co-glycolic acid) (PLGA). This complex compound additionally radiolabeled with lutetium-177 (^177^Lu), was found to exhibit desirable effects when intravenously injected in athymic mice with a subcutaneous breast tumor model (MDA-MB-231 cells). More importantly, the interaction between each pharmacophore of the compound was synergistic, as the inhibition of breast tumor growth was highest compared to its structural parts, i.e., PLGA(PTX) and ^177^Lu-BN-PLGA administered alone.

Camptothecin (CPT) and its analogs are widely used as topoisomerase I inhibitors, thus reducing the growth of several tumors [119]. Although CPT is unstable in human plasma and exhibits a high degree of toxicity [120,121,122], this compound has been linked with BN (particularly [D-Tyr^6^,β-Ala^11^,Phe^13^,Nle^14^]BN-) via a carbamate linker with a built-in nucleophile-associated releasing group (an ethylenediamine-containing linker (L1) and an N-methylethylenediamine (L2)) to generate a potent drug candidate for the treatment of BN receptor-containing tumor cells [123,124]. Among these various compounds, only intraperitoneal or subcutaneous CPT-L2-BA3 showed its high cytotoxic effect against NCI-H1299 non-small cell lung cancer (NSCLC) cells and expressed its affinity toward all three types of BN receptors [123]. In contrast, its analogs, such as D-Phe-CPT-L2-BA3, had a much-reduced cytotoxic effect both in vitro and in vivo [124]. Furthermore, considering the poor plasma stability of CPT, CPT-L2-BA3 had enhanced stability, as its half-life was equal to 30 min in mouse plasma and exceeded the value for GRP of <5 min [124].

Another example of BN effectively used as a building element of a hybrid was proposed by Aranda-Lara et al. [125], who designed and synthesized a radiolabeled BN-based drug with modified folate, which was found to be essential for cancer cells during DNA synthesis and repair [126], while its receptors (FR) were overexpressed in all clinical breast cancer subtypes [127]. As a consequence, Lys1(α,γ-Folate)Lys3(99mTc-EDDA/HYNIC)-BN 1–14) occurred to display high recognition by BB2 and the human FR with the IC50 value < 10 nmol/L [125].

BN was also used to construct a specific BN-conjugated nanosystem, including liposomes and micelles. One excellent example is the work of Accardo et al. [128], who demonstrated liposomes to contain as a single entity a BN(7–14) peptide fragment, the DTPA chelating agent, a hydrophobic molecule with two C18 alkyl chains, and polyethylene glycol (PEG) spacers. Another publication reported solid lipid nanoparticles containing BN with doxorubicin [129]. These novel conjugates showed desirable anticancer activity in vivo and excellent cytotoxicity in vitro using the PC3 prostate cancer cell line or the MCF-7/MDR breast cancer cell line, respectively (all the compounds were administered intravenously).

## 4. Conclusions

The hybridization of two biologically active substances into one moiety, a method classified as one of the most promising for improving the pharmacological properties of a drug, reducing drug–drug interactions, and resulting in negligible toxicity and low-cost preclinical studies, has now gained much attention. Of the several different compounds considered useful structural building blocks for such new chimeric molecules, bombesins appear to be low on the list of potential candidates, as there are still a small number of reports on the synthesis of hybrid compounds involving BN or its structural analogs. Indeed, the literature in the field of BN-based hybrid compounds is relatively sparse. To date, few such structures have been presented. However, since preliminary in vitro or in vivo studies indicate the superiority of BN hybrids over a single drug, especially in terms of their efficacy and safety profile, it seems that BN and its analogs should attract much attention as a potent active pharmacophore. It is worth noting that the new proposed hybrid structures containing BN may be designed not only for oncological purposes but also as potential therapeutics in other pathologies where the role of BNs is already well documented. This may be important since BNs have been found to interact with several other target receptors in addition to BN receptors, as mentioned earlier.

Therefore, this article aims to encourage researchers to focus on bombesins and indicates that a hybrid approach should also be firmly applied to bombesins and the BN receptor family.

## Figures and Tables

**Table 1:** Similarities between amino acid sequences of bombesin and mammalian bombesin-like peptides, including binding affinities toward the bombesin receptor family.

Peptide	Amino Acid Sequence	Affinities at Bombesin Receptor Subtypes * IC_50_ [nM]		
		BB1	BB2	BB3
Bombesin (BN)	pGlu-Gln-Arg-Leu-Gly-Asn-Gln-**Trp-Ala-Val-Gly-His-Leu-Met**-NH_2_	1.77 ± 0.04	0.07 ± 0.01	>3000
Gastrin-releasing peptide (GRP)	Ala-Pro-Val-Ser-Val-Gly-Gly-Gly-Thr-Val-Leu-Ala-Lys-Met-Tyr-Pro-Arg-Gly-Asn-His-**Trp-Ala-Val-Gly-His-Leu-Met**-NH_2_	148.0 ± 8.00	0.17 ± 0.01	>3000
Neuromedin B	Gly-Asn-Leu-Trp-Ala-Thr-**Gly-His-Phe-Met**-NH_2_	0.052 ± 0.003	50.1 ± 2.50	>3000

* Binding affinities were determined using Balb-3T3 cells, which were stable and expressed human BB1, BB2 or BB3 receptors. These values are taken from [8]. BB1—a mammalian NMB-preferring receptor (neuromedin B receptor); BB2—a GRP-preferring (GRP receptor); BB3—an orphan receptor designated as bombesin receptor subtype-3. Bold and underlines indicate differences and similarities between the peptides.

**Table 2:** Structural similarities and differences between amphibian bombesin-like peptides including binding affinities toward the bombesin receptor family.

Peptide	Amino Acid Sequence	Affinities at Bombesin Receptor Subtypes * IC_50_ [nM]		
		BB1	BB2	BB3
Bombesin (BN)	pGlu-Gln-Arg-Leu-Gly-Asn-Gln-**Trp-Ala-Val-Gly-His-Leu-Met**-NH_2_	34	4	>10,000
Alytensin	pGlu-Gly-Arg-Leu-Gly-Thr-Gln-**Trp-Ala-Val-Gly-His-Leu-Met**-NH_2_	460	62	>10,000
Litorin	pGlu-Gln-**Trp-Ala-Val-Gly-His-Phe-Met**-NH_2_	7	6	>10,000
Phyllolitorin	pGlu-Leu-**Trp-Ala-Val-Gly-Ser-Phe-Met**-NH_2_	47	240	>10,000
[Leu^8^]-phyllolitorin	pGlu-Leu-**Trp-Ala-Val-Gly-Ser-Leu-Met**-NH_2_	372	295	>3000
[Thr^5^,Leu^8^]-phyllolitorin (R-phyllolitorin)	pGlu-Leu-**Trp-Ala-Thr-Gly-Ser-Leu-Met**-NH_2_	unknown	unknown	unknown
Ranatensin	pGlu-Val-Pro-Gln-**Trp-Ala-Val-Gly-His-Phe-Met**-NH_2_	13	2	>10,000

* Binding affinity values were taken from [8,12]. BB1—a mammalian NMB-preferring receptor (neuromedin B receptor); BB2—a GRP-preferring (GRP receptor); BB3—an orphan receptor designated as bombesin receptor subtype-3. Bold and underlines indicate differences and similarities between the peptides.

## References

[1] Kerru N., Singh P., Koorbanally N., Raj R., Kumar V. (2017). Recent advances (2015–2016) in anticancer hybrids. Eur. J. Med. Chem..

[2] Kleczkowska P. (2022). Chimeric structures in mental illnesses—“Magic” molecules specified for complex disorders. Int. J. Mol. Sci..

[3] Cosledan F., Fraisse L., Pellet A., Guillou F., Mordmuller B., Kremsner P.G., Moreno A., Mazier D., Maffrand J.-P., Meunier B. (2008). Selection of a trioxaquine as an antimalarial drug candidate. Proc. Natl. Acad. Sci. USA.

[4] Kamath P.R., Sunil D., Ajees A.A., Pai K.S.R., Das S. (2015). Some new indole–coumarin hybrids: Synthesis, anticancer and Bcl-2 docking studies. Bioorg. Chem..

[5] Klingenstein R., Lober S., Kujala P., Godsave S., Leliveld S.R., Gmeiner P., Peters P.J., Korth C. (2006). Tricyclic antidepressants, quinacrine and a novel, synthetic chimera thereof clear prions by destabilizing detergent resistant membrane compartments. J. Neurochem..

[6] Pradhan T.K., Katsuno T., Taylor J.E., Kim S.H., Ryan R.R., Mantey S.A., Donohue P.J., Weber H.C., Sainz E., Battey J.F. (1998). Identification of a unique ligand which has high affinity for all four bombesin receptor subtypes. Eur. J. Pharmacol..

[7] Erspamer V., Erspamer G.F., Inselvini M. (1970). Some pharmacological actions of alytesin and bombesin. J. Pharm. Pharmacol..

[8] Uehara H., Gonzales N., Sancho V., Mantey S., Nuche-Berenuer B., Pradhan T., Coy D.H., Jensen R.T. (2011). Pharmacology and selectivity of various natural and synthetic bombesin related peptide agonists for human and rat bombesin receptors differs. Peptides.

[9] McDonald T.J., Jörnvall H., Nilsson G., Vagne M., Ghatei M., Bloom S.R., Mutt V. (1979). Characterization of a gastrin releasing peptide from porcine non-antral gastric tissue. Biochem. Biophys. Res. Commun..

[10] Taché Y., Vale W., Rivier J., Brown M. (1980). Brain regulation of gastric secretion: Influence of neuropeptides. Proc. Natl. Acad. Sci. USA.

[11] Chen X.J., Sun Y.G. (2020). Central circuit mechanisms of itch. Nat. Commun..

[12] Jensen R.T., Battey J.F., Spindel E.R., Benya R.V. (2008). International Union of Pharmacology. LXVIII. Mammalian bombesin receptors: Nomenclature, distribution, pharmacology, signaling, and functions in normal and diseases states. Pharmacol. Rev..

[13] Moody T.W., Merali Z. (2004). Bombesin-like peptides and associated receptors within the brain: Distribution and behavioral implications. Peptides.

[14] Plamondon H., Merali Z. (1997). Anorectic action of bombesin requires receptor for corticotropin-releasing factor but not for oxytocin. Eur. J. Pharmacol..

[15] Deschodt-Lanckman M., Robberecht P., De Neef P., Lammens M., Christophe J. (1976). In vitro action of bombesin and bombesin-like peptides on amylase secretion, calcium efflux, and adenylate cyclase activity in the rat pancreas: A comparison with other secretagogues. J. Clin. Investig..

[16] Erspamer V., Improta G., Melchiorri P., Sopranzi N. (1974). Evidence of cholecystokinin release by bombesin in the dog. Br. J. Pharmacol..

[17] Ghatei M.A., Jung R.T., Stevenson J.C., Hillyard C.J., Adrian T.E., Lee Y.C., Christofides N.D., Sarson D.L., Mashiter K., MacIntyre I. (1982). Bombesin: Action on gut hormones and calcium in man. J. Clin. Endocrinol. Metab..

[18] Rozengurt E. (1990). Bombesin stimulation of mitogenesis. Specific receptors, signal transduction, and early events. Am. Rev. Respir. Dis..

[19] Rozengurt E., Sinnett-Smith J. (1983). Bombesin stimulation of DNA synthesis and cell division in cultures of Swiss 3T3 cells. Proc. Natl. Acad. Sci. USA.

[20] Wiedermann C.J., Ruff M.R., Pert C.B. (1988). Bombesin-like peptides: Neuropeptides with mitogenic activity. Brain Behav. Immun..

[21] Assimakopoulos S.F., Alexandris I.H., Scopa C.D., Mylonas P.G., Thomopoulos K.C., Georgiou C.D., Nikolopoulou V.N., Vagianos C.E. (2005). Effect of bombesin and neurotensin on gut barrier function in partially hepatectomized rats. World J. Gastroenterol..

[22] Assimakopoulos S.F., Scopa C.D., Zervoudakis G., Mylonas P.G., Georgiou C., Nikolopoulou V., Vagianos C.E. (2005). Bombesin and neurotensin reduce endotoxemia, intestinal oxidative stress, and apoptosis in experimental obstructive jaundice. Ann. Surg..

[23] Brown D.R., Gillespie M.A. (1988). Actions of centrally administered neuropeptides on rat intestinal transport: Enhancement of ileal absorption by angiotensin II. Eur. J. Pharmacol..

[24] Guarini S., Tagliavini S., Bazzani C., Bertolini A. (1989). Bombesin reverses bleeding-induced hypovolemic shock, in rats. Life Sci..

[25] Merali Z., Johnston S., Zalcman S. (1983). Bombesin-induced behavioral changes: Antagonism by neuroleptics. Peptides.

[26] Baroni A., Perfetto B., Canozo N., Braca A., Farina E., Melito A., De Maria S., Carteni M. (2008). Bombesin: A possible role in wound repair. Peptides.

[27] Sun Y.G., Chen Z.F. (2007). A gastrin-releasing peptide receptor mediates the itch sensation in the spinal cord. Nature.

[28] Lee H., Ko M.C. (2015). Distinct functions of opioid-related peptides and gastrin-releasing peptide in regulating itch and pain in the spinal cord of primates. Sci. Rep..

[29] Green P.G. (2005). Gastrin-releasing peptide, substance P and cytokines in rheumatoid arthritis. Arthritis Res. Ther..

[30] Moody T.W., Carney D.N., Cuttitta F., Quattrocchi K., Minna J.D. (1985). High affinity receptors for bombesin/GRP-like peptides on human small cell lung cancer. Life Sci..

[31] Rashidy-Pour A., Razvani M.E. (1998). Unilateral reversible inactivations of the nucleus tractus solitarius and amygdala attenuate the effects of bombesin on memory storage. Brain Res..

[32] Flood J.F., Morley J.E. (1988). Effects of bombesin and gastrin-releasing peptide on memory processing. Brain Res..

[33] Ferreira L.B.T., Oliveira S.L.B., Raya J., Esumi A., Hipolide D.C. (2017). Bombesin administration impairs memory and does not reverse memory deficit caused by sleep deprivation. Behav. Brain Res..

[34] Mountney C., Sillberg V., Kent P., Anisman H., Merali Z. (2006). The role of gastrin-releasing peptide on conditioned fear: Differential cortical and amygdaloid responses in the rat. Psychopharmacology.

[35] Mountney C., Anisman H., Merali Z. (2008). Effects of gastrin-releasing peptide agonist and antagonist administered to the basolateral nucleus of the amygdala on conditioned fear in the rat. Psychopharmacology.

[36] Sakamoto H., Matsuda K., Zuloaga D.G., Hongu H., Wada E., Wada K., Jordan C.L., Breedlove S.M., Kawata M. (2008). Sexually dimorphic gastrin releasing peptide system in the spinal cord controls male reproductive functions. Nat. Neurosci..

[37] DeWitt R.C., Wu Y., Renegar K.B., King B.K., Li J., Kudsk K.A. (2000). Bombesin recovers gut-associated lymphoid tissue and preserves immunity to bacterial pneumonia in mice receiving total parenteral nutrition. Ann. Surg..

[38] Dal-Pizzol F., Pons Di Leone L., Ritter C., Martins M.R., Reinke A., Gelain D.P., Zanotto-Filho A., de Souza L.F., Andrades M., Frediani Barberio D. (2006). Gastrin-releasing peptide receptor antagonists effects on an animal model of sepsis. Am. J. Respir. Crit. Care Med..

[39] Ohki-Hamazaki H., Iwabuchi M., Maekawa F. (2005). Development and function of bombesin-like peptides and their receptors. Int. J. Dev. Biol..

[40] Bedard T., Mountney C., Kent P., Anisman H., Merali Z. (2007). Role of gastrin-releasing peptide and Neuromedin B in anxiety and fear-related behavior. Behav. Brain Res..

[41] Matusiak D., Glover S., Nathaniel R., Matkowskyj K., Yang J., Benya R.V. (2005). Neuromedin B and its receptor are mitogens in both normal and malignant epithelial cells lining the colon. Am. J. Physiol. Gastrointest. Liver Physiol..

[42] Moody T.W., Jensen R.T., Garcia L., Leyton J. (2000). Nonpeptide Neuromedin B receptor antagonists inhibit the proliferation of C6 cells. Eur. J. Pharmacol..

[43] Von Schrenck T., Heinz-Erian P., Moran T., Mantey S.A., Gardner J.D., Jensen R.T. (1989). Neuromedin B receptor in esophagus: Evidence for subtypes of bombesin receptors. Am. J. Physiol..

[44] Fleming M.S., Ramos D., Han S.B., Zhao J., Son Y.-J., Luo W. (2012). The majority of dorsal spinal cord gastrin releasing peptide is synthesized locally whereas neuromedin B is highly expressed in pain- and itch-sensing somatosensory neurons. Mol. Pain..

[45] Mishra S.K., Holzman S., Hoon M.A. (2012). A nociceptive signaling role for neuromedin B. J. Neurosci..

[46] Wan L., Jin H., Liu X.Y., Jeffry J., Barry D.M., Shen K.F., Peng J.H., Liu X.T., Jin J.H., Sun Y. (2017). Distinct roles of NMB and GRP in itch transmission. Sci. Rep..

[47] Boughton C.K., Patel S.A., Thompson E.L., Patterson M., Curtis A.E., Amin A., Chen K., Ghatei M.A., Bloom S.R., Murphy K.G. (2013). Neuromedin B stimulates the hypothalamic–pituitary–gonadal axis in male rats. Regul. Pept..

[48] Todman M.G., Han S.K., Herbison A.E. (2005). Profiling neurotransmitter receptor expression in mouse gonadotropin-releasing hormone neurons using green fluorescent protein-promoter transgenics and microarrays. Neuroscience.

[49] Zhao S., Guo Z., Xiang W., Wang P. (2021). The neuroendocrine pathways and mechanisms for the control of the reproduction in female pigs. Anim. Reprod..

[50] Park H.J., Kim M.K., Kim Y., Kim H.J., Bae S.K., Bae M.K. (2021). Neuromedin B modulates phosphate-induced vascular calcification. BMB Rep..

[51] Trayhurn P., Wood I.S. (2004). Adipokines: Inflammation and the pleiotropic role of white adipose tissue. Br. J. Nutr..

[52] Kroog G.S., Jensen R.T., Battey J.F. (1995). Mammalian bombesin receptors. Med. Res. Rev..

[53] Jensen R.T., Moody T.W., Kastin A.J. (2013). Bombesin-related peptides. Handbook of Biologically Active Peptides.

[54] Hildebrand P., Lehmann F.S., Ketterer S., Christ A.D., Stingelin T., Beltinger J., Gibbons A.H., Coy D.H., Calam J., Larsen F. (2001). Regulation of gastric function by endogenous gastrin releasing peptide in humans: Studies with a specific gastrin releasing peptide receptor antagonist. Gut.

[55] Hirschowitz B.J., Gibson R.G. (1978). Stimulation of gastrin release and gastric secretion: Effect of bombesin and a nonapeptide in fistula dogs with and without fundic vagotomy. Digestion.

[56] Lenz J.H., Forquignon I., Druge G., Greten H. (1989). Effects of neuropeptides on gastric acid and duodenal bicarbonate secretions in freely moving rats. Regul. Pept..

[57] Varner A.A., Modlin I.M., Walsh J.H. (1981). High potency of bombesin for stimulation of human gastrin release and gastric acid secretion. Regul. Pept..

[58] Beltran B., Barrachina M.D., Mendez A., Quintero E., Esplugues J.V. (1999). Synthesis of nitric oxide in the dorsal motor nucleus of the vagus mediates the inhibition of gastric acid secretion by central bombesin. Br. J. Pharmacol..

[59] Bertaccini G., Erspamer V., Impicciatore M. (1973). The actions of bombesin on gastric secretion of the dog and the rat. Br. J. Pharmacol..

[60] Martinez V., Tache Y. (2000). Bombesin and the brain-gut axis. Peptides.

[61] Gonzalez N., Moody T.W., Igarashi H., Ito T., Jensen R.T. (2008). Bombesin-related peptides and their receptors: Recent advances in their role in physiology and disease states. Curr. Opin. Endocrinol. Diabetes Obes..

[62] Hirooka A., Hamada M., Fujiyama D., Takanami K., Kobayashi Y., Oti T., Katayama Y., Sakamoto T., Sakamoto H. (2021). The gastrin-releasing peptide/bombesin system revisited by a reverse-evolutionary study considering Xenopus. Sci. Rep..

[63] Fathi Z., Corjay M.H., Shapira H., Wada E., Benya R., Jensen R., Viallet J., Sausville E.A., Battey J.F. (1993). BRS-3: A novel bombesin receptor subtype selectively expressed in testis and lung carcinoma cells. J. Biol. Chem..

[64] Wada E., Way J., Lebacq-Verheyden A.M., Battey J.F. (1990). Neuromedin B and gastrin-releasing peptide mRNA are differentially distributed in the rat nervous system. J. Neurosci..

[65] Wang H., Bian J., Chen Z., Miao Y., Li W. (2011). A novel bombesin-like peptide from skin of Rana shuchinae. Mol. Biol. Rep..

[66] De Sousa N.A., Marani M.M., Lopes A.L.F., Silva E.M., Barbosa E.A., Vasconcelos A.G., Kuzniewski F.T.B., Lustosa S.S., Gomes K.P., Colugnati D.B. (2022). BR-bombesin: A novel bombesin-related peptide from the skin secretion of the Chaco tree frog (Boana raniceps) with physiological gastric effects. Amino Acids.

[67] Miao Y., Li W., Duan L., Xiao Y. (2010). A bombesin-like peptide from skin of Sanguirana varians. Comp. Biochem. Physiol. B Biochem. Mol. Biol..

[68] Endean R., Erspamer V., Erspamer G.F., Improta G., Melchiorri P., Negri L., Sopranzi N. (1975). Parallel bioassay of bombesin and litorin, a bombesin-like peptide from the skin of Litoria aurea. Br. J. Pharmacol..

[69] Rrivier C., Rivier J., Vale W. (1978). The effect of bombesin and related peptides on prolactin and growth hormone secretion in the rat. Endocrinology.

[70] Modlin I.M., Lamers C.B.H., Walsh J.H. (1981). Stimulation of canine pancreatic polypeptides gastrin and gastric acid secretion by rantensin, litorin, bombesin on a peptide and substance P. Regul. Pept..

[71] Mitsuma T., Nogimori T., Sun D.H., Chaya M. (1986). Litorin (bombesin family) inhibits thyrotropin secretion in rats. Exp. Clin. Endocrinol..

[72] de Caro G., Massi M., Micossi L.G., Perfumi M. (1984). Drinking and feeding inhibition by ICV pulse injection or infusion of bombesin, ranatensin and litorin to rats. Peptides.

[73] Gibbs J., Kulkosky P.J., Smith G.P. (1981). Effects of peripheral and central bombesin on feeding behavior of rats. Peptides.

[74] Kulkosky P.J., Sanchez M.R., Marrinan D.A. (1992). Bombesin reduces alcohol choice in nutritive expectancy and limited-access procedures. Alcohol.

[75] Geller R.G., Govier W.C., Pisano J.J., Tanimura T., Van Clineschmidt B. (1970). The action of ranatensin, a new polypeptide from amphibian skin, on the blood pressure of experimental animals. Br. J. Pharmacol..

[76] Howard J.M., Jensen R.T., Gardner J.D. (1985). Bombesin-induced residual stimulation of amylase release from mouse pancreatic acini. Am. J. Physiol..

[77] Jensen R.T., Gardner J.D. (1981). Identification and characterization of receptors for secretagogues on pancreatic acinar cells. Federation Proc..

[78] Cline M.A., Fouse D.N., Prall B.C. (2008). Central and peripheral alytesin cause short-term anorexigenic effects in neonatal chicks. Neuropeptides.

[79] Negri L., Improta G., Briccardo M., Melchiorri P. (1988). Phyllolitorins: A new family of bombesin-like peptides. Ann. N. Y. Acad. Sci..

[80] King K.A., Torday J.S., Sunday M.E. (1995). Bombesin and [Leu8]phyllolitorin promote fetal mouse lung branching morphogenesis via a receptor-mediated mechanism. Proc. Natl. Acad. Sci. USA.

[81] Broccardo M., Cadamone A. (1985). The effects of a new amphibian peptide, Leu8phyllolitorin, on thermoregulation in the rat. Peptides.

[82] Merali Z., Johnston S., Sistek J. (1985). Role of dopaminergic system(s) in mediation of the behavioral effects of bombesin. Pharmacol. Biochem. Behav..

[83] Van Wimersma Greidanus T.B., Maigret C., Torn M., Ronner E., Van der Kracht S., Van der Wee N.J., Versteeg D.H. (1989). Dopamine D-1 and D-2 receptor agonists and antagonists and neuropeptide-induced excessive grooming. Eur. J. Pharmacol..

[84] Van Wimersma Greidanus T.B., van de Brug F., de Bruijckere L.M., Pabst P.H., Ruesink R.W., Hulshof R.L., van Berckel B.N., Arissen S.M., de Koning E.J., Donker D.K. (1988). Comparison of bombesin-, ACTH-, and beta-endorphin-induced grooming. Antagonism by haloperidol, naloxone, and neurotensin. Ann. N. Y. Acad. Sci..

[85] Zhu X.Z., Ji X.Q., Wu S.X., Zou G. (1991). Sulpiride attenuates ranatensin-M-induced antinociception. Zhongguo Yao Li Xue Bao.

[86] Laskowska A.K., Szudzik M., Ścieżyńska A., Komorowski M., Szűcs E., Gombos D., Bączek B., Lipka- Miciuk J., Benyhe S., Kleczkowska P. (2022). The role of a natural amphibian skin-based peptide, ranatensin, in pancreatic cancers expressing dopamine D2 receptors. Cancers.

[87] Merali Z., Graitson S., MacKay J.C., Kent P. (2013). Stress and eating: A dual role for bombesin-like peptides. Front. Neurosci..

[88] Roesler R., Luft T., Oliveira S.H.S., Farias C.B., Almeida V.R., Quevedo J., Dal Pizzol F., Schroder N., Izquierdo I., Schwartsmann G. (2006). Molecular mechanisms mediating gastrin-releasing peptide receptor modulation of memory consolidation in the hippocampus. Neuropharmacology.

[89] Buhot M.C. (1997). Serotonin receptors in cognitive behaviors. Curr. Opin. Neurobiol..

[90] Chaouloff F., Berton O., Mormède P. (1999). Serotonin and stress. Neuropsychopharmacology.

[91] Merali Z., Bedard T., Andrews N., Davis B., McKnight A.T., Gonzalez M.I., Pritchard M., Kent P., Anisman H. (2006). Bombesin receptors as a novel anti-anxiety therapeutic target: BB1 receptor actions on anxiety through alterations of serotonin activity. J. Neurosci..

[92] Pinnock R.D., Reynolds T., Woodruff G.N. (1994). Different types of bombesin receptors on neurons in the dorsal raphe nucleus and the rostral hypothalamus in rat brain slices in vitro. Brain Res..

[93] Piqueras L., Taché Y., Martínez V. (2003). Somatostatin receptor type 2 mediates bombesin-induced inhibition of gastric acid secretion in mice. J. Physiol..

[94] Liu X.Y., Ginosar Y., Yazdi J., Hincker A., Chen Z.F. (2019). Cross-talk between human spinal cord μ-opioid receptor 1Y isoform and gastrin-releasing peptide receptor mediates opioid-induced scratching behavior. Anesthesiology.

[95] Gmerek D.E., Cowan A. (1988). Role of opioid receptors in bombesin-induced grooming. Ann. N. Y. Acad. Sci..

[96] Bungo T., Ando R., Kawakami S.-I., Ohgushi A., Shimojo M., Masuda Y., Furuse M. (2000). Central bombesin inhibits food intake and the orexigenic effect of neuropeptide Y in the neonatal chick. Physiol. Behav..

[97] Chang M.M., Leeman S.E., Niall H.D. (1971). Amino-acid sequence of substance P. Nat. New Biol..

[98] Grimsholm O., Rantapää-Dahlqvist S., Forsgren S. (2005). Levels of gastrin-releasing peptide and substance P in synovial fluid and serum correlate with levels of cytokines in rheumatoid arthritis. Arthritis Res. Ther..

[99] Regoli D., Dion S., Rhaleb N.E., Drapeau G., Rouissi N., Orléans-Juste P. (1988). Receptors for neurokinins, tachykinins, and bombesin: A pharmacological study. Ann. N. Y. Acad. Sci..

[100] Sakurada T., Manome Y., Katsumata K., Uchiumi H., Tan-No K., Sakurada S., Kisara K. (1992). Naloxone-reversible effect of spantide on the spinally mediated behavioural response induced by neurokinin-2 and -3 receptor agonists. Naunyn Schmiedebergs Arch. Pharmacol..

[101] Moura E.G., Santos C.V., Santos R.M., Pazos-Moura C.C. (1999). Interaction between substance P and gastrin-releasing peptide on thyrotropin secretion by rat pituitary in vitro. Braz. J. Med. Biol. Res..

[102] Laskowska A.K., Kleczkowska P. (2022). Anticancer efficacy of endo- and exogenous potent ligands acting at dopaminergic receptor-expressing cancer cells. Eur. J. Pharmacol..

[103] Bruna-Larenas T., Gomez-Jeria J.S. (2012). A DFT and semiempirical model-based study of opioid receptor affinity and selectivity in a group of molecules with a morphine structural core. Int. J. Med. Chem..

[104] Lipinski C.A., Lombardo F., Dominy B.W., Feeney P.J. (2001). Experimental and computational approaches to estimate solubility and permeability in drug discovery and development settings. Adv. Drug Deliv. Rev..

[105] Akiyama T., Tominaga M., Takamori K., Carstens M.I., Carstens E. (2014). Role of spinal bombesin-responsive neurons in nonhistaminergic itch. J. Neurophysiol..

[106] Sun Y.-G., Zhao Z.-Q., Meng X.-L., Yin J., Liu X.-Y., Chen Z.-F. (2009). Cellular basis of itch sensation. Science.

[107] Liu S., Yang H., Wan L., Cai H., Li S., Li Y., Cheng J., Lu X. (2011). Enhancement of cytotoxicity of antimicrobial peptide magainin II in tumor cells by bombesin-targeted delivery. Acta Pharmacol. Sin..

[108] Lehmann J., Retz M., Sidhu S.S., Suttmann H., Sell M., Paulsen F., Harder J., Untergger G., Stockle M. (2006). Antitumor activity of the antimicrobial peptide magainin II against bladder cancer cell lines. Eur. Urol..

[109] Cruciani R.A., Barker J.L., Zasloff M., Chen H.C., Colamonici O. (1991). Antibiotic magainins exert cytolytic activity against transformed cell lines through channel formation. Proc. Natl. Acad. Sci. USA.

[110] Serafin P., Kowalczyk P., Stefanucci A., Laskowska A.K., Zawadzka M., Kramkowski K., Kleczkowska P. (2023). Evaluation of antimicrobial activities against various E. coli strains of a novel hybrid peptide—LENART01. Molecules.

[111] Xu X., Lai R. (2015). The chemistry and biological activities of peptides from amphibian skin secretions. Chem. Rev..

[112] Stein C. (2013). Targeting pain and inflammation by peripherally acting opioids. Front. Pharmacol..

[113] Kroll C., Mansi R., Braun F., Dobitz S., Maecke H.R., Wennemers H. (2013). Hybrid bombesin analogues: Combining an agonist and an antagonist in defined distances for optimized tumor targeting. J. Am. Chem. Soc..

[114] Santos-Cuevas C.L., Ferro-Flores G., Arteaga de Murphy C., Ramirez F.M., Luna-Gutierez M.A., Pedraza-Lopez M., Garcia-Becerra R., Ordaz-Rosado D. (2009). Design, preparation, in vitro and in vivo evaluation of (99m)Tc-N2S2-Tat(49-57)-bombesin: A target-specific hybrid radiopharmaceutical. Int. J. Pharm..

[115] Begum A.A., Toth I., Moyle P.M. (2019). Gastrin-releasing peptide receptor-targeted hybrid peptide/phospholipid pDNA/siRNA delivery systems. Nanomedicine.

[116] Nagy A., Armatis P., Cai R.Z., Szepeshazi K., Halmos G., Schally A.V. (1997). Design, synthesis, and in vitro evaluation of cytotoxic analogs of bombesin-like peptides containing doxorubicin or its intensely potent derivative, 2-pyrrolinodoxorubicin. Proc. Natl. Acad. Sci. USA.

[117] Kiaris H., Schally A., Nagy A., Sun B., Armatis P., Szepeshazi K. (1999). Targeted cytotoxic analogue of bombesin/gastrin-releasing peptide inhibits the growth of H-69 human small-cell lung carcinoma in nude mice. Br. J. Cancer.

[118] Gibbens-Bandala B., Morales-Avila E., Ferro-Flores G., Santos-Cuevas C., Melendez-Alafort L., Trujillo-Nolasco M., Ocampo-Garcia B. (2019). ^177^Lu-Bombesin-PLGA (paclitaxel): A targeted controlled-release nanomedicine for bimodal therapy of breast cancer. Mater. Sci. Eng. C.

[119] Yang S., Zhu J., Lu Y., Liang B., Yang C. (1999). Body distribution of camptothecin solid lipid nanoparticles after oral administration. Pharm. Res..

[120] Mi Z., Burke T.G. (1994). Differential interactions of camptothecin lactone and carboxylate forms with human blood components. Biochemistry.

[121] Gottlieb J.A., Luce J.K. (1972). Treatment of malignant melanoma with camptothecin (NSC-100880). Cancer Chemother. Rep..

[122] Muggia F.M., Creaven P.J., Hansen H.H., Cohen M.H., Selawry O.S. (1972). Phase I trial of weekly and daily treatment with camptothecin (NSC-100880): Correlation with preclinical studies. Cancer Chemother. Rep..

[123] Moody T.W., Mantey S.A., Pradhan T.K., Schumann R., Nakagawa T., Martinez A., Fusilier J., Coy D.H., Jensen R.T. (2004). Development of high affinity camptothecin-bombesin conjugates that have targeted cytotoxicity for bombesin receptor-containing tumor cells. J. Biol. Chem..

[124] Moody T.W., Sun L.C., Mantey S.A., Pradhan T., Mackey L.V., Gonzales N., Fuselier J.A., Coy D.H., Jensen R.T. (2006). In vitro and in vivo antitumor effects of cytotoxic camptothecin-bombesin conjugates are mediated by specific interaction with cellular bombesin receptors. J. Pharmacol. Exp. Ther..

[125] Aranda-Lara L., Ferro-Flores G., Ramirez F.M., Ocampo-Garcia B., Santos-Cueva C., Diaz-Nieto L., Isaac-Olive K. (2016). Improved radiopharmaceutical based on 99mTc-Bombesin-folate for breast tumour imaging. Nucl. Med. Commun..

[126] Pieroth R., Paver S., Day S., Lammersfeld C. (2018). Folate and its impact on cancer risk. Curr. Nutr. Rep..

[127] Zhang Z., Li P., Chen H., Wei B., Xiao X., Da J., Skinner K., Hicks D.G., Bu H., Tang P. (2014). Folate receptor [alpha] associated with triple-negative breast cancer and poor prognosis. Arch. Pathol. Lab. Med..

[128] Accardo A., Salsano G., Morisco A., Aurilio M., Parisi A., Maione F., Cicala C., Tesauro D., Aloj L., De Rosa G. (2012). Peptide-modified liposomes for selective targeting of bombesin receptors overexpressed by cancer cells: A potential theranostic agent. Int. J. Nanomed..

[129] Wang C., Sun X., Wang K., Wang Y., Yang F., Wang H. (2016). Breast cancer targeted chemotherapy based on doxorubicin-loaded bombesin peptide modified nanocarriers. Drug Deliv..

